# Digital contact tracing and exposure notification: ethical guidance for trustworthy pandemic management

**DOI:** 10.1007/s10676-020-09566-8

**Published:** 2020-10-21

**Authors:** Robert Ranisch, Niels Nijsingh, Angela Ballantyne, Anne van Bergen, Alena Buyx, Orsolya Friedrich, Tereza Hendl, Georg Marckmann, Christian Munthe, Verina Wild

**Affiliations:** 1 International Center for Ethics in the Sciences and Humanities, University of Tübingen, Tübingen, Germany; 2 Institute of Ethics, History and Theory of Medicine, Ludwig-Maximilians-University Munich, Munich, Germany; 3 Centre for Biomedical Ethics, National University of Singapore, Singapore, Singapore; 4 Institute for History and Ethics in Medicine, Technical University Munich, Munich, Germany; 5 Institute of Philosophy, FernUniversität Hagen, Hagen, Germany; 6 Department of Philosophy, Linguistics and Theory of Science, University of Gothenburg, Gothenburg, Sweden

**Keywords:** Contact tracing apps, Covid-19, Public trust, Public health, Health apps, mHealth

## Abstract

There is growing interest in contact tracing apps (CT apps) for pandemic management. It is crucial to consider ethical requirements before, while, and after implementing such apps. In this paper, we illustrate the complexity and multiplicity of the ethical considerations by presenting an ethical framework for a responsible design and implementation of CT apps. Using this framework as a starting point, we briefly highlight the interconnection of social and political contexts, available measures of pandemic management, and a multi-layer assessment of CT apps. We will discuss some trade-offs that arise from this perspective. We then suggest that public trust is of major importance for population uptake of contact tracing apps. Hasty, ill-prepared or badly communicated implementations of CT apps will likely undermine public trust, and as such, risk impeding general effectiveness.

## Introduction: the rise of digital contact tracing

Digital technologies are increasingly being discussed and implemented for Covid-19 pandemic management and as tools for easing restrictive measures, such as lockdowns (Mello and Wang 2020; Ting et al. 2020). Due to the high penetration rate of smartphones, there has been a huge interest in mobile phone data as a source for public health research and measures (Oliver et al. 2020). To track the spread of the virus, in Europe and elsewhere, network operators share (anonymized and aggregated) phone location data. Apple and Google, two leading providers of smartphone operating systems, release data to show mobility trends in countries and selected regions (Apple; Google 2020). In addition, a range of new mobile phone based applications (“apps”), sometimes lumped together under the term “COVID-19 apps”, have been rolled out recently or are being under development by private as well as public actors (Sharma and Bashir 2020; Privacy International 2020; Woodhams 2020; GDPRhub 2020).

These apps may serve a variety of functions: provide users with Covid-19-related information, monitor people in quarantine, trace movements, or give users rapid warning of potential exposure to SARS-CoV-2 (GDPRhub 2020; Rimpiläinen et al. 2020). Frequently, mobile phone apps are designed to fulfil more than just one purpose, e.g. symptom checkers could generate data which might also be used for epidemiological modelling, monitoring the virus spread or to evaluate public health measures. Available apps differ widely regarding data use (e.g. self-reported, geolocation data, proximity tracing), data sources (e.g. GPS, Bluetooth), data handling (decentralized or centralized), as well as data protection (anonymization or pseudonymization) (Woodhams 2020).

*Proximity* or *contact tracing apps* (CT apps) have gained notable attention so far. CT apps notify users if they have been in proximity to confirmed infected people and propose next steps (e.g. self-isolation, testing). A vital distinction to be made here is between apps that collect data on—in principle—identifiable individuals in a centralised database (‘centralised’ variants) and those that function by use of encrypted identifiers that connect individual users to each other (‘decentralised’ variants). Only the first allows ‘contact tracing’ in the stricter sense when individuals and encounters are retrospectively identified by a third party. The second variant warns users in the case of contact with infected individuals (i.e. exposure notification), but does not allow a centralized tracing of possible infection chains. Both variants, contact tracing and exposure notification, can play an important role in a digitally supported pandemic management strategy.

Since analogous contact tracing is comparatively slow, resource intense and lacks reliability, digital proximity tracing has been proposed as a complementary tool to indicate possible transmission chains that analogous contact tracing might miss or take a longer time to identify. One study suggests that CT apps could, in theory, effectively decrease virus transmission by enabling targeted testing or quarantine, and thus avoid mass confinements or lockdowns (Ferretti et al. 2020). Informing or identifying potential spreaders earlier could reduce pre-symptomatic transmission, i.e. before an infected person shows symptoms. This might also support micromanagement after lifting restrictive pandemic control measures or during future infection waves. Until today, however, little is known about the effectiveness and efficiency of CT apps in real-world settings, and whether or not they could also have negative effects on pandemic management, or expose individuals to ethical downsides, such as lack of data protection.

As of August 2020, a wide range of CT apps is being used or under development globally, from Algeria to Vietnam (Howell O'Neill et al. 2020). Singapore pioneered a Bluetooth based open-source technology named Bluetrace, which underpins the *TraceTogether app* (TraceTogether 2020). In Europe, after a joint attempt to establish a pan-European “privacy-preserving approach” for CT (PEPP-PT) has seemingly failed, various countries have rolled out their own proximity tracing apps. Initially, PEPP-PT and a centralized database were considered as the preferred framework for CT apps, but massive criticism (e.g. Joint Statement 2020) has led some policy makers to switch to a decentralised approach (Busvine and Rinke 2020). However, European countries are divided on the question whether to rely on centralized (e.g. France) or decentralized (e.g. Germany) data management, making interoperability between different frameworks difficult. By now, the authorities of many European countries like Austria, Belgium, Denmark, Germany, Italy, Ireland and Switzerland opted for a decentralised approach based on a joint API from Apple and Google (Howell O'Neill et al. 2020). There are plans from the European Commission to build a gateway to allow cross-border exchange of information between these national CT apps.

Development of CT apps is not only promoted by public agencies but often relies on public–private partnerships with relevant corporate actors. Notably, Apple and Google have collaborated to develop a joint contact tracing framework which is also founded on decentralized data management (Apple and Google 2020). Such efforts are important to guarantee interoperability between different smartphone systems and allow building efficient CT apps. However, due to this dependency, commercial companies are gaining a wide-ranging influence on the national strategies for digital contract tracing; e.g. the Apple and Google framework is only of limited use for countries which have opted for a centralized architecture for CT apps like France (Scott et al. 2020). Furthermore, the use of the framework is restricted to only one tracing app per country (Gurman and De Vynck 2020).

CT apps may prove to be valuable public health tools, but they also raise significant concerns (Gasser et al. 2020; Lucivero et al. 2020). As part of the Covid-19 pandemic response, advisory bodies, NGOs, and expert initiatives have interrogated the ethical aspects of digital surveillance technologies, including CT apps (e.g. AlgorithmWatch 2020; Amnesty International et al. 2020; Chaos Computer Club 2020; Human Rights Watch 2020; Swiss National Advisory Commission on Biomedical Ethics 2020; WHO 2020). The first ethical frameworks for digital tools in the context of Covid-19 have been proposed (Mello and Wang 2020; Gasser et al. 2020; Lucivero et al. 2020; Kahn et al. 2020; Morley et al. 2020; Parker et al. 2020), and the European Commission (2020) has drafted various recommendations and guidelines for digital contact tracing in the EU.

This paper focuses on ethical considerations for responsible development, design and implementation of effective and justifiable CT apps in pandemic management strategies. It considers legal and digital ethical concerns in a broader framework of public health ethics as well as related pragmatic and procedural considerations. It provides a framework for ethical analysis of concrete proposals, and suggests that to strengthen trustworthiness, policy makers need to be sensitive to the multi-faceted complexities of public health decision making.

## Ethical framework for decision-making on the use of CT apps

The viability of CT apps as a useful pandemic-response measure, depends on a complex interplay of criteria, such as pragmatic assumptions about effectiveness, the likelihood of public health benefit, technological specifications, legal requirements etc. To minimise the risk of adverse outcomes, ethical standards should guide and complement the process of development (ethics by design), implementation, use, and evaluation of CT apps. Rather than asking general questions on the moral acceptability of CT apps, the crucial question is: “*What specific interventions, if any, may be justified under what conditions?”* Inspired by ethical frameworks for big data in health and research, developed by the SHAPES initiative (Xafis et al. 2020), and other normative frameworks for digital health technologies (Marckmann 2020) and pandemic management (Thompson et al. 2006), we propose relevant substantive values (which evaluate the outcome of measures) and procedural values (which guide decision-making) as well as corresponding questions, which should be considered in response to these requirements (Table 1).

**Table 1:** Ethical framework for CT apps: substantive and procedural values

Substantive values	Guiding questions
Public health benefit	Is the pandemic situation such that contact tracing activity is motivated from a public health standpoint? Is the general use of the CT app likely to enhance the effectiveness of contact tracing measures? Is the technological make-up of the app such that it can actually produce public health benefit? Is the pool of potential users who are willing to use a CT app large enough for epidemiological effectiveness?
Harm minimisation	Are CT apps the least harmful way of obtaining the desired benefits? Are CT apps easy to use and do they minimise confusion or stress by design? Has the risk of self- and social stigma effects, implicated by an elevated focus on one’s or others’ health status been considered and mitigated? Are safeguards in place to mitigate the vulnerability of and harm to marginalized groups from CT apps and related public health and security measures? Are potential, harmful social effects related to the app (widespread anxiety, ineffective quarantines etc.) adequately considered?
Privacy	Are measures in place for data protection and against data loss or misuse?Are data security authorities involved? Is data parsimony guaranteed and access to non-essential personal data minimised? Are the most privacy-preserving solutions (e.g. no real-time data, anonymization) prioritised? Is collection of the tracing-data temporary (e.g. will it be deleted after a certain, specified amount of time)? Is data sharing for other purposes excluded? Are appropriate cyber-resilience measures in place?
Justice	Has accessibility and availability been maximised Are benefits and burdens of CT apps equally distributed among the population? Will discrimination of vulnerable and structurally disadvantaged population groups be prevente? Are there measures to safely include marginalized groups or ‘digital immigrants’, without exacerbating their vulnerability? Will resulting scientific knowledge and insights be freely shared for the public good? Are different levels of digital literacy considered in app design?
Liberty/autonomy	Are users informed about possible consequences of CT app use? Are CT apps used voluntarily? Is there proper user consent for data use? Are users able to withdraw consent? Are there measures to avoid de-facto mandatory use, e.g. by restricting access to public or work space with CT apps? Are CT apps the least liberty-compromising measures compared to alternative strategies to pandemic management? Are there alternatives for those who choose not to participate in CT apps?
Solidarity	Are there measures to avoid negative effects on solidarity, e.g. by not imposing overly disproportionate burdens on specific groups? Has consideration been given to whether negative attitudes towards people who do not use the app may feed into practices of victim blaming?
Stewardship	Are effects of CT apps on existing infrastructure considered (e.g. encourage or strengthen power asymmetries, or market monopolies)? Are safeguards against function creep, i.e. the use beyond the purpose of the technology, in place? Are there strategies against malicious, fake CT apps? Are measures and policies reversible? Are CT apps embedded in robust regulatory frameworks? Are safeguards and oversight mechanisms in place? Are strategies in place to limit duration and end measures (sunset provisions)?

Procedural values	Guiding questions
Transparency	Are technological solutions and frameworks sufficiently transparent (e.g. open source)? Are purposes, objectives, as well as limitations of CT apps and measures clearly named and communicated? Are actors and possible stakes behind the CT apps transparent? Can CT apps be subject to an audit?
Proportionality	Are social, and moral costs of CT apps proportionate to the pandemic threat and the expected effectiveness of using the app? Is the cost-effectiveness of the CT app positive compared to alternative pandemic management strategies? Are financial costs proportionate to the expected public health benefits?
General trustworthiness	Are democratic procedures in place to guide decision making? Can population uptake be assumed? Do stated objectives of CT apps align with proposed measures?
Reasonableness	Is the proposed solution epidemiologically sound? Are the underlying considerations and models scientifically valid? Is there sufficient evidence that the CT app meets technical standards of reliance? Is the pandemic situation of a stage that makes contact tracing a priority from a public health standpoint? Is the app embedded in and of added value to a robust public health strategy with sufficient resources to test, trace and treat?
Accountability	Is it clear who can be held to account in the case of adverse outcomes, such as harm, infringements of rights or lack of effectiveness? Is there oversight of CT apps by legitimate governmental agencies and independent oversight bodies?
Consistency	Are CT measures and policies based on the same legal and ethical standards as other accepted measures of pandemic management? Are policies consistent with legal frameworks? Do strategies fit with local or national demands for pandemic management?
Engagement	Are there possibilities for the broader public to participate in decision making? Has input from relevant stakeholders (e.g. public authorities, health departments) been considered?
Reflexivity	Are there alternative strategies of contract tracing prepared if CT apps turn out to be inefficient and are there strategies in place to reverse decisions? Have the potential effects of CT apps on data monopolies be considered in decision making Are there research initiatives in place to evaluate the efficiency of CT apps?

The list of considerations provides a sketch of the complex set of criteria relevant to assessing CT apps as ethically justifiable public health tools. We neither claim that the list is complete, nor do we think that a responsible policy-making process should necessarily address all of them. On the contrary, it is highly unlikely that a solution would satisfy all these demands. Not only is there a significant lack of available data and real-world experience regarding CT technologies, *all* pandemic management strategies will involve several trade-offs. But acknowledging the ethical values and specific questions can help during development, implementation and evaluation of CT apps in order to find ethically appropriate solutions. In what follows, we will describe some of the complexities in implementing CT apps (cf. Nijsingh et al. 2020).

## The landscape of justifying CT apps

**Fig. 1:**
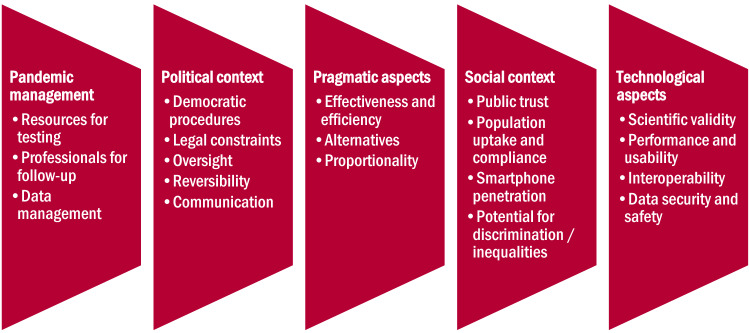
Layers of assessment: To assess CT apps, the interplay between technological aspects and socio-political contexts needs to be considered

Considering the wide variety of mobile applications being developed in the context of the Covid-19 pandemic, it is crucial to distinguish between different apps, their functions, purposes, and performance. The value of mobile applications being developed in the context of the Covid 19 pandemic essentially depends on specific pandemic contexts and factors such as the social and political environment, how CT apps are integrated into a comprehensive strategy of pandemic management, as well as possible and available alternatives (Fig. 1).

Notably, and as we will demonstrate, the implementation of digital contact tracing may involve moral costs. In some countries, apps and other mobile based surveillance measures are imposed on people, leading to an infringement of privacy rights (Human Rights Watch 2020). Even without compulsion, CT apps can have severe consequences for social values: worries range from issues of data protection, to possible stigmatization of patients, social justice concerns, or function creep (Woodhams 2020; Hart et al. 2020).

Nevertheless, risks that cannot be easily mitigated or avoided could still be acceptable, considering the severity of a pandemic situation, the importance of effective contact tracing to manage it, and the scope of established measures to stop virus transmission. To assess whether a certain CT app is justified, its use needs to be compared to available alternative strategies. From this perspective, infringements associated with a possible loss of privacy and risks related to an effective CT app may appear justifiable in light of the enormous costs in terms of welfare, liberty and health outcomes of either letting the virus run its course or maintaining comprehensive restrictions or lockdowns (Schaefer and Ballantyne 2020).

To make a case in favour of a CT app, however, several conditions must be met. Sufficient societal need and potential effectiveness need to be demonstrated, and ethical risks sufficiently mitigated in order to demonstrate proportionality. In addition, such evaluation and decision-making needs to demonstrate procedural fairness, with transparency and opportunity for potentially concerned parties to voice concerns. Finally, the balance of reasons for and against needs to be superior to alternative solutions or strategies. Here, again, context matters. For a CT app scheme to be worth its costs and risks, a society needs to be in a pandemic stage, in which contact tracing is a priority. This may depend both on the pattern of (community) transmission, and the healthcare capacity of this country relative to the transmission pattern. If a society is not in such a state, no app will be able to promote better contact tracing.

In addition, the utility of CT apps largely depends on broader public health measures beyond digital technologies. For CT apps to contribute to an effective public health strategy, sufficient staffing of public health services as well as reliable infrastructures (e.g. for testing and for quarantine) are needed. To avoid false positive self-reports, health departments or other institutions need to confirm infection status of users. For ‘centralised’ CT apps, the data generated by the app needs to be collected and analysed in a meaningful and cost-effective way (from a public health perspective) in relation to a set of justified effective tracing actions that are thereby being facilitated (i.e. eased or made possible by the app data). For ‘decentralised’ apps, additional efforts of analogous contact tracing are necessary, because possible transmission chains are not tracked in a way to be accessible for health authorities. All CT apps require well-organised institutional efforts.

### Enhancing effectiveness

Little is known about the effectiveness of contact tracing apps in the real-world setting (Anderson 2020). Even for countries with a high penetration rate of proximity tracing technologies such as Iceland, the contribution of CT apps to suppressing the pandemic has been questioned (Johnson 2020). Besides the risks of false positives (which can impose burden on unaffected individuals) and false negatives (which may lead to a false sense of security), the implementation of an ineffective app has opportunity costs: wasting time and resources, undercutting other solutions and leading to wrong political decisions. This may result in a sub-optimal approach to pandemic control, leading to higher morbidity and mortality and greater economic damage.

It is also crucial to view the value of a CT app in regards to the quality of information produced by it: Mobile phones are not well equipped for contact tracing of individuals. Bluetooth signals, which are central for the now widely used approach supported by Apple and Google, only allow a rough estimation between devices (Leith and Farrell 2020). The same is true if location data (GPS) is used. Apps that would rely on user-generated subjective information are also likely to produce false predictions that could affect particular tracing policies. This concerns both false positives and false negatives. As such, incorrect information will rather compromise than support particular public health measures, as well as health care systems more generally, and scarce resources may be wasted, or used suboptimally.

By contrast, CT apps that appear to be effective in tracing individuals, may raise more severe privacy concerns (Baumgärtner et al. 2020). It has been reported from South Korea, where multi-source tracing and tracking technologies are being used (GDPRhub 2020), that information was so detailed as to allow re-identification of individuals (Zastrow 2020). Hence, the values of effectiveness and privacy need to be carefully balanced in digital public health measures. For example, while infringements on individual rights or liberties could be justified to secure health benefits, measures always need to be proportionate and aim for careful balance between competing values and considerations.

### Population uptake

Effectiveness does not only presuppose a favourable context in terms of a suitable pandemic stage and accompanying interventions, but also sufficient uptake. For CT apps to offer a meaningful contribution to pandemic management, a large part of the population needs access to compatible mobile technologies (e.g. newer smartphones or beacons), install and set up the app, and be willing and able to use tools correctly.

A study from the UK has estimated that to stop the pandemic on its own, around 80% of smartphone users (more than 50% of population overall) would have to use a CT app (Hinch et al. 2020), i.e. a user rate comparable to WhatsApp or Facebook Messenger in some European countries. As mentioned, so far the highest penetration rate of CT apps in the world has been reported from Iceland, where almost 40% of the overall population downloaded a CT app. For Singapore’s much heralded CT app, less than a quarter of the population are using this tool (TraceTogether 2020). At the point of writing in early August 2020 Germany had introduced a CT app less than two months ago, and download numbers had reached more than 16 million, approximately 20% of the overall population (Robert Koch Institut 2020). A lower adoption rate still has some positive effect for targeted testing and quarantine (Howell O'Neill 2020; Hinch et al. 2020). Nevertheless, population uptake is a bottleneck for success of these digital technologies.

Predicting future uptake of CT apps is difficult and depends on various factors, such as the penetration range rate of digital technologies in a society, the possibility to download and use the app on different types of smartphones, the credibility of institutions offering these solutions, and viable solutions for ethical concerns such as data security. Recent surveys have been inconclusive about the possible uptake in different countries. A study showed a high level of support (around 80%) for CT apps in countries such as the UK, Germany, France and the US (Milsom et al. 2020), while other surveys from the US and Germany came to a less optimistic conclusion (Anderson and Auxier 2020; COVID-19 Snapshot Monitoring 2020). The available data also show that some aspects could reduce the acceptability of CT apps: these include concerns about further continuation of surveillance after the pandemic and data security (Anderson and Auxier 2020).

One way to increase uptake is, of course, to force people to download and use CT apps. Mandatory use of disease surveillance tools and possible moral obligations to comply with them are being discussed (Lucivero et al. 2020; Parker et al. 2020; Schaefer and Ballantyne 2020). Coercion, however, adds ethical downsides of liberty restrictions that are seen as substantial in a liberal democratic context, and thereby complicates the justification of a CT app policy. Moreover, compulsory measures may undermine public trust and create incentives for cheating (Floridi 2020), necessitating even more forceful steps to secure the benefits of the policy. As a consequence, then these benefits need to be even more pronounced and certified in order to create a potential for the policy to be proportional.

For this reason, CT app programs based on voluntary use with a good uptake appear preferable. But this assumes strong public trust in the apps and the program (Ienca and Vayena 2020; Parker et al. 2020). Trust, however, must build on trustworthiness, and thus needs to be backed up by responsible design and corresponding policies. Such “well founded” credence (Parker et al. 2020) also remains a strong indicator that choices are self-determined and, thus, in line with democratic values.

Meanwhile, reports from China and other nations have already shown that digital measures utilised in the Covid-19 pandemic response have been used for mass surveillance (Woodhams 2020; Human Rights Watch 2020) and that there might be plans to massively extend the use of newly established apps even after pandemic (Davidson 2020). In some countries such as Sweden or the Netherlands, the launch of CT apps has been postponed or even cancelled due to weak data security and doubts about effectiveness and concerns on the legality of apps that process sensitive personal information (Wassens 2020; Hagberg 2020). Such evidence might have already fuelled public mistrust in CT apps in other nations, especially in societies, in which trust in science and governance is limited. For countries like Germany, public outreach by the political representation regarding the introduction of different apps has created confusion (Barker 2020). Internationally, CT apps have already become the subject of conspiracy theories, fake news, and scams.

From the perspective of liberal values, citizens should ideally support CT apps because they have (justified) faith in public health measures and, thus, freely choose to utilise disease surveillance technologies. This, however, does not rule out some measures to increase population uptake (Floridi 2020): encouragement, campaigning, nudges and even some stronger forms of incentives could be justified to increase adoption rates. Possible benefits should be equally accessible for most citizens without disproportionate burdens, and negative incentives must not be so severe as to render CT apps de facto compulsory, for example by limiting access to essential infrastructures (Lucivero et al. 2020; Morley et al. 2020). Incentives can also create new risks, e.g. owing to users’ psychological responses to the information regarding user-surroundings and related health risks disclosed by a particular app. A privacy infringing, unfair or burdensome app may trigger negative responses, particularly if it is perceived as being imposed upon the public.

### Public trust

Uptake depends in part on the level of trust in agencies responsible for development, marketing, and distribution of CT apps, on solving issues e.g. of data protection or stigmatization, but also on the usefulness and performance of digital proximity tracing itself. Since using CT apps could have adverse consequences for individuals, for example by requiring tests and imposing isolation measures, demonstrated effectiveness and validity of CT apps will be a major factor for population uptake. Trust, however, cannot be quickly established, or specifically for just one public health intervention (Ward 2017). It is a long-term endeavour and requires constant efforts to uphold it, e.g. through transparent communication and participatory elements in health care planning.

This raises a pragmatic dilemma regarding the factor of trust: on the one hand, the effectiveness of CT apps is uncertain. On the other hand, digital proximity tracing essentially depends on population uptake and user adherence. Broad scepticism about the effectiveness of digital contract tracing could eventually become a self-fulfilling prophecy.

This pragmatic dilemma must therefore also be incorporated into ethical considerations. For if the probability of uptake and thus of effective pandemic control with the app is too small, the risks and moral costs of the app could be too high. For an ethically appropriate introduction of an app, that also maintains or increases well-founded trust, the functions, goals, possible chances, and risks associated with specific CT apps must be communicated clearly, as well as the measures taken to mitigate the risks. The same goes for disclosure of conflicts of interest and the procedural management of state-business relationships linked to commissions of technological development and procurement of technical products.

This last aspect becomes especially important if the decision is to adopt one particular national CT app solution and policy, meaning that private developers will be in serious competition to win the race for a state contract. To increase app uptake, focusing efforts on one single CT app with just one (or a limited number of) clearly defined purpose(s) and broad support from political and health institutions may be crucial. To prevent confusion and loss of trustworthiness, there may then be good reasons to restrict privately offered CT apps, or to institute mandatory quality assurance authorisation in order to ensure that pandemic management is not undermined by business ventures.

The importance of trustworthiness of technologies and policies for earning sustainable public trust also means that it is important to prevent false expectations. For instance, simplistic “solutionism”, i.e. the belief that pandemic challenges could be managed by technological fixes alone, must be avoided.

Public decision-making on pandemic policies including decision making on CT apps, requires a structured framework to work through these ethical considerations. Such a framework can play a vital role in increasing transparency of made decisions, as well as the trustworthiness of (and trust in) policies and technical solutions.

## Conclusion

Based on our analysis, we conclude the following points for consideration:

- The Covid-19 pandemic cannot be solved by technological means alone. Digital proximity tracing is not a panacea in the Covid-19 pandemic response, but could become a valuable component in a comprehensive strategy. Thus, it is imperative to have appropriate public health measures and infrastructures in place before and while implementing CT apps.
- To ensure effectiveness and user-friendliness, there should only be a limited number of CT apps or, ideally, only one platform. Reducing the functionality of apps, i.e. only one clear objective per app, seems advisable. While a joint, pan-European platform, allowing interoperability between different CT apps is warranted, diverging requirements need to be considered.
- Given the inevitable risks for privacy and the potential impact on individual liberty, especially related to the centralized CT apps, there should be a reasonable expectation of population benefit of CT apps prior to their large-scale applications. Effectiveness and benefits must be evaluated alongside the implementation.
- The ubiquitous presence of risks necessitates a thorough and prudent approach. A particular focus on temporary measures is warranted. While science and policy have been confronted with deep uncertainty during the Covid-19 pandemic, strategies must be carefully chosen, risks mitigated and measures reversible. Uncertainties on the benefits of digital CT limit the set of legitimate pandemic response policies and actions. Without sufficiently clear evidence of effectiveness, jeopardizing the rights or liberties of (some parts of) the population cannot be justified.
- Trust is essential in public health decision-making in general, and Covid-19 CT apps in particular. Policies, recommendations and public health measures should be part of a broader endeavour to win and maintain trust in public health measures. Well-founded trust requires taking seriously the ethical complexities relating to the implementation of CT apps as well as being transparent about the inevitable trade-offs that are being made. Communicating goals and functions as well as possible benefits, risks, and limitations of CT apps clearly and early can play a crucial role in preventing squandering trust and misconceptions.
